# Carcinogenesis of PIK3CA

**DOI:** 10.1186/1897-4287-11-5

**Published:** 2013-06-15

**Authors:** Sidra German, Hafiz Muhammad Aslam, Shafaq Saleem, Aisha Raees, Tooba Anum, Arsalan Ahmad Alvi, Abdul Haseeb

**Affiliations:** 1 Final year student of Dow University of Health Sciences, Karachi, Pakistan; 2 Flat #14, 3rd floor, Rafiq Mansion, Cambell road, Off Arambagh, Karachi, Pakistan

## Abstract

PIK3CA is the most frequently mutated oncogene in human cancers. PIK3CA is phosphatidylinositol-4,5-bisphosphate 3-kinase, catalytic subunit alpha. It controls cell growth, proliferation, motility, survival, differentiation and intracellular trafficking. In most of human cancer alteration occurred frequently in the alpha isoform of phosphatidylinositol 3 kinase. PIK3CA mutations were most frequent in endometrial, ovarian, colorectal, breast, cervical, squamous cell cancer of the head and neck, chondroma, thyroid carcinoma and in cancer family syndrome. Inhibition of PI3K signaling can diminish cell proliferation, and in some circumstances, promote cell death. Consequently, components of this pathway present attractive targets for cancer therapeutics. A number of PI3K pathway inhibitors have been developed and used. PI3K inhibitors (both pan-PI3K and isoform-specific PI3K inhibitors), dual PI3K-mTOR inhibitors that are catalytic site inhibitors of the p110 isoforms and mTOR (the kinase component of both mTORC1 and mTORC2), mTOR catalytic site inhibitors, and AKT inhibitors are the most advanced in the clinic. They are approved for the treatment of several carcinomas.

## Findings

PIK3 was discovered by Lewis Cantley and his colleagues [1]. PIK3CA is phosphatidylinositol-4,5-bisphosphate 3-kinase, catalytic subunit alpha. The pathway, with oncogene PIK3CA and tumor suppressor PTEN (gene), is implicated in insensitivity of cancer tumors to insulin and IGF1, in calorie restriction. PIK3 controls cell growth, proliferation, motility, survival, differentiation and intracellular trafficking [2]. There are two subunits 85 kDa regulatory subunit and a 110 kDa catalytic subunit. Gene of PIK3 is located on chromosome 3q26.3 [3]. The encoded protein represents the catalytic subunit, which uses ATP to phosphorylate PtdIns, PtdIns4P and PtdIns (4,5)P2. This gene is kinases in PI3K family. PIK3 classify into three classes on the basis of primary structure, regulation and in vitro lipid substrate specificity [4]. Class I PI3K is responsible for cell growth, proliferation and survival. It consists of two subclasses: Ia - dimeric components, comprised of catalytic subunits: p110α, p110β, p110δ, associated with p85 regulatory subunit and subclass Ib, which is heterodimers consisting of p110γ catalytic subunit, connected with p101 regulatory subunit for transmission of signals from receptor tyrosine kinase –RTK(i.e. EGFR, PDGFR). In most of human cancers, regulation of this signal transduction pathway is frequently disrupted by alterations of PI3K pathway [5,6].

In most of human cancer the gene which is frequently altered is that encodes the alpha isoform of phosphatidylinositol 3 kinase [6]. PIK3CA mutations were most frequent in endometrial (21%), ovarian (17%), colorectal (17%), breast 14%), cervical (13%), and squamous cell cancer of the head and neck (9%) [7]. In Cloves Syndrome mutations occur in PIK3CA gene in a range of 3-30%. Cloves mainly caused by post zygotic activating mutation of PIK3CA [8].

With regard to mutation frequencies, PIK3CA is the most frequently mutated oncogene in human cancers. Generally pik3ca gene is deregulated by PTEN gene (tumor suppressor gene). In many tumors PTEN mutation leads to hyperactivity of PIK3CA oncogene [9]. Diverse PIK3CA mutations activate lipid kinase activity hence changing confirmation of cytosolic membranes. This up regulates enzymatic activity serving as a common mutated gene in cancers.

In thyroid carcinoma PIK3 pathway is involved, and activation of this pathway is through stimulatory molecules or through loss of inhibitory molecules [10]. In one study it is seen that PIK3CA mutation is not common rather its amplification is common and may be mechanism in activation of PIK3/akt in some thyroid cancer [11]. Activation of PIK3/akt is frequently involved in benign thyroid adenoma (BTA), follicular thyroid cancer (FTC) and anaplastic thyroid cancer (ATC) and progression from BTA to FTC to ATC [11]. Mutation in PIK3CA is also seen in the pathogenesis of thyroid cancer relatively common in anaplastic thyroid carcinoma. In Middle Eastern papillary thyroid carcinoma, synergistic effect of PIK3 and BRAF suggest their role in tumorogensis [12].

It is proved that mutation of PIK3CA occur in significant number of human glioblastoma multiforme [13].

Similarly high expression of PIK3CA is associated with increased chances colorectal metastasis. PIK3CA inhibitors may be beneficial in the treatment of colorectal cancer and decreases invasiveness of melanoma cells [14].

Cancer family Syndrome is a genetic disorder in which genetic mutations occur in or more genes predisposed the effected individual to the development of cancer and may also cause the early onset of tumor [15]. Main culprit in causing Cowden Syndrome is PTEN mutation but 8 of 91(8.8%) unrelated Cowden syndrome individual without germ line mutations carries 10 germ line PICK3CA mutation (7 missense, 1 non-sense and 2 indels) [16]. Analysis at exon 1,7,9 and 20 of the PIK3CA gene revealed somatic mutations in 21% (8 out of 39) of familial adenomatous polyposis invasive carcinoma, 21% (7 out of 34) of Hereditary non polyposis colorectal invasive carcinoma, 15% (8 of 52%) of sporadic invasive carcinoma and 14% (7 out of 50) of sporadic colorectal metastasis in liver. Mutation in Familial adenomatous polyposis and HNPCC predominantly occur in kinase domain (exon 20), while majority of mutations in sporadic cases occurred in Helical domain (exon 9) [17]. In another study it was identified that in HNPCC, mutation in PIK3CA was identified in 14% tumor while over expression in 59% of tumor [18].

Exon 9 and 20 of the PIK3CA gene were analyzed for clear cell adenocarcinoma, it was found that somatic mutations of PIK3CA gene was detected in 10/23(43%) and in all cases the type of mutation was H1047R in the kinase domain. Findings suggest that mutation of PIK3CA gene occur in putative pre cursor lesion of CCA ( Clear cell adenocarcinoma) [19].

In chondroma there is involment of 3p263-q 29 gene causing loss of PIK3CA gene [20]. In patients of primary endometrial carcinoma, mutation occur in PIK3CA pathway occur in 172(16.2%) tumor, mostly they were high grade tumor [21]. Son et al. reported that in 40% prostatic carcinomas, PIK3CA amplification occurs in 13% and PIK3CA mutation in 3% tumor [22].

Among patients with mutated-PIK3CA colorectal cancers, regular use of aspirin after diagnosis was associated with superior colorectal cancer–specific survival and overall survival but In contrast, among patients with wild-type PIK3CA*,* regular use of aspirin after diagnosis was not associated with colorectal cancer–specific survival [23].

The prognostic markers may have another role in predicting and guiding the clinical treatment of cancer patients by allowing the identification of patients suited to current therapies. In this era of molecularly targeted therapy, inhibitors and antibodies targeting specific molecules are vigorously being developed, and some have been demonstrated to be effective in clinical settings.

The PI3K/Akt pathway is one of the most important signaling pathways in human carcinogenesis. Importantly, PIK3CA amplification could aberrantly activate the PI3K/Akt signaling pathway. Inhibition of PI3K signaling can diminish cell proliferation, and in some circumstances, promote cell death. Consequently, components of this pathway present attractive targets for cancer therapeutics. A number of PI3K pathway inhibitors have been developed and are being evaluated in preclinical studies and in early clinical trials. Rapamycin analogs, such as temsirolimus and everolimus that specifically inhibit mTORC1 are the most advanced in the clinic, and they are approved by Food and drug administration for the treatment of advanced renal cell carcinoma. Other PI3K pathway inhibitors include PI3K inhibitors (both pan-PI3K and isoform-specific PI3K inhibitors), dual PI3K-mTOR inhibitors that are catalytic site inhibitors of the p110 isoforms and mTOR (the kinase component of both mTORC1 and mTORC2), mTOR catalytic site inhibitors, and AKT inhibitors. Not only do these agents have the capacity to inhibit cancer cell proliferation and survival signals as described above, but they may also impact tumor angiogenesis, metastasis, and metabolism [2].

## Competing interest

Authors declared that they have no competing interest.

## Authors’ contributions

SG, HMA and SS did manuscript drafting while AR, TA, AAA and AH did critical review. All authors give approval of final version.

## Authors’ information

Sidra German: Final Year students of Dow Medical College, Dow University of Health Sciences. Hafiz Muhammad Aslam: Final Year students of Dow Medical College, Dow University of Health Sciences. Shafaq Saleem: Final Year students of Dow Medical College, Dow University of Health Sciences. Aisha Raees: Final Year students of Dow Medical College, Dow University of Health Sciences. Tooba Anum: Final Year students of Dow Medical College, Dow University of Health Sciences. Arsalan Ahmad Alvi: Final Year students of Dow Medical College, Dow University of Health Sciences. Abdul Haseeb: Final Year students of Dow Medical College, Dow University of Health Sciences.
